# "Body-In-The-Loop": Optimizing Device Parameters Using Measures of Instantaneous Energetic Cost

**DOI:** 10.1371/journal.pone.0135342

**Published:** 2015-08-19

**Authors:** Wyatt Felt, Jessica C. Selinger, J. Maxwell Donelan, C. David Remy

**Affiliations:** 1 Department of Mechanical Engineering, University of Michigan, Ann Arbor, MI, United States of America; 2 Department of Biomedical Physiology and Kinesiology, Simon Fraser University, Burnaby, BC, Canada; Nankai University, CHINA

## Abstract

This paper demonstrates methods for the online optimization of assistive robotic devices such as powered prostheses, orthoses and exoskeletons. Our algorithms estimate the value of a physiological objective in real-time (with a body “in-the-loop”) and use this information to identify optimal device parameters. To handle sensor data that are noisy and dynamically delayed, we rely on a combination of dynamic estimation and response surface identification. We evaluated three algorithms (*Steady-State Cost Mapping*, *Instantaneous Cost Mapping*, and *Instantaneous Cost Gradient Search*) with eight healthy human subjects. *Steady-State Cost Mapping* is an established technique that fits a cubic polynomial to averages of steady-state measures at different parameter settings. The optimal parameter value is determined from the polynomial fit. Using a continuous sweep over a range of parameters and taking into account measurement dynamics, *Instantaneous Cost Mapping* identifies a cubic polynomial more quickly. *Instantaneous Cost Gradient Search* uses a similar technique to iteratively approach the optimal parameter value using estimates of the local gradient. To evaluate these methods in a simple and repeatable way, we prescribed step frequency via a metronome and optimized this frequency to minimize metabolic energetic cost. This use of step frequency allows a comparison of our results to established techniques and enables others to replicate our methods. Our results show that all three methods achieve similar accuracy in estimating optimal step frequency. For all methods, the average error between the predicted minima and the subjects’ preferred step frequencies was less than 1% with a standard deviation between 4% and 5%. Using *Instantaneous Cost Mapping*, we were able to reduce subject walking-time from over an hour to less than 10 minutes. While, for a single parameter, the Instantaneous Cost Gradient Search is not much faster than Steady-State Cost Mapping, the Instantaneous Cost Gradient Search extends favorably to multi-dimensional parameter spaces.

## Introduction

Advances in rehabilitation technology have led to a growing class of computer-controlled devices that provide powered assistance for human motion. These include powered protheses, orthoses, and exoskeletons [1]. The behavior of these devices can often be tuned via a set of control parameters. For instance, the BiOM powered ankle prosthesis has 11 parameters governing its stiffness, the power and timing of the actuated push-off, and other functionality [2]. The powered prosthesis for trans-femoral amputees developed at Vanderbilt had 42 virtual-stiffness parameters at one point in its development [3]. Although high numbers of parameters make devices highly adaptable, they also make the selection of proper parameter values more difficult. One might hope to select appropriate parameters settings using models and simulations, but parameter settings selected in this way are not always optimal when evaluated experimentally [4]. Offline models cannot fully capture the variability of end-users nor the ways that users will interact with different parameter settings. This makes an *a priori* selection of parameters for a specific end-user a challenge. For this reason, final parameter selections are almost always experimentally. That is, the process is driven by experimental observations of the user interacting with the device at different parameter settings [2]. The methods for tuning parameters to a particular end-user are often based on heuristics. The tuning may involve a trial-and-error process that is driven by subjective evaluations [4]. This is similar to the process used by a prosthetist when aligning a conventional passive prosthesis [5]. The process requires expert knowledge, is time consuming, and research has shown that the subjectivity of these methods makes them unreliable [6, 7]. It would be highly desirable to automate the tuning process to remove subjectivity and enable assistive devices to continue to adapt even after the initial fitting.

The development of such an automated tuning process begins with the definition of a suitable physiological objective. An optimization algorithm that is able to quantify this objective can automatically compare parameter choices and identify those that are better. Possible examples of physiological objectives include the reduction of energy spent by the user, improved user comfort, or the enforcement of a desired user training effort. Generally, none of these objectives can be measured directly. Instead, they must be estimated from related indicators, such as from oxygen consumption, muscle activation, heart rate, or joint motion. The relationship between these indicators and the physiological objective can be complex and is often concealed by unrelated effects, such as noise or other disturbances. To reconstruct the objective, we might have to combine information from multiple sensors and take into account delays and other dynamic effects. In this paper we focus our work on using metabolic energetic cost as the physiological objective. We estimate this cost by using oxygen consumption measured at the mouth as the related indicator. In our methods, we compensate for the dynamics of energy conversion in the body and we employ algorithms that are robust against the large amounts of noise that are typical for respiratory measurements. Naturally, these methods are very specific to energetic cost. However, similar ideas can be applied to a wide range of physiological objectives as well as to different indicators and sensor modalities.

Metabolic energetic cost is commonly used to characterize and improve device performance [8–11]. It is a measure of the energy being consumed by a user to sustain an activity. As the user of an assistive device is able to rely more on the device to perform the necessary work, the energy required of a user goes down [12]. In this way, energetic cost provides a clear way to characterize the quality of assistance being provided. For example, Malcolm et al. [13] demonstrated that measurements of energetic cost can be used to identify the energetically optimal timing setting of a powered ankle-foot orthosis. Deviations away from this optimal setting resulted in increased user effort. There is also evidence that, in many situations, humans seek to minimize their energetic cost in unassisted walking. Research has shown that a subject’s choice of walking speed, step-frequency and step width will minimize the energy required to walk a given distance [14–17]. In addition, using metabolic effort as the objective might allow us to capture some other important characteristics of providing beneficial assistance. For example, if the wrong parameter setting of an assistive device causes discomfort or pain, the user might start fighting the device or engage in other compensatory actions. Through this mechanism, discomfort and other issues will likely lead to an increase in energy consumption and are thus accounted for in measures of energetic cost.

The use of measurements of energetic cost is challenging because they are sparsely sampled, noisy, and dynamically delayed [18]. The most practical measures during locomotion come from measuring the volumes of oxygen and carbon-dioxide in inspired and expired air. These measures are used to approximate the consumption rates of chemical fuels in the body [19]. The sampling rate of these measures corresponds to the breath-rate of the subject. During light exercise, a typical breath rate of 20 breaths-per-minute would result in a sampling rate of 0.3Hz [20]. Variability in breath volumes results in noisy measurements with a Gaussian distribution and a signal to noise ratio of approximately four [18, 21]. Additionally, the measures do not always reflect the user’s current level of effort. Rather, the effect of changes in effort appears gradually over time. For example, if a sitting subject were asked to stand and begin walking, it would take over a minute for the energetic cost measures to rise and plateau at the value corresponding to walking [20].

When looking to identify optimal parameter settings, the difficulties of respiratory measurements are typically addressed by averaging the effects of many steady-state breaths. To characterize the energetic cost at a given parameter setting, researchers will often wait three minutes to allow the measures to plateau at steady-state values and then average measurements over an additional three minutes while the subject continues the activity [20]. This process is repeated with several parameter settings to approximate the relationship between the energetic cost and the parameter through a curve-fitting process. The minimum of this curve is taken as a proxy for the optimal parameter settings [13–15]. We refer to this method as *Steady-State Cost Mapping*. *Mapping*, refers to the fact that this method seeks to fit a curve or surface over a large number of parameter settings to estimate the shape of the overall energy-parameter relationship. *Steady-State Cost* is the energetic cost averaged at a single parameter setting after some waiting time to avoid any effects of the dynamic delay and noise.

With only a single parameter to tune, Steady-State Cost Mapping can be quite effective. The time required to collect each data point, however, greatly limits the broader applicability of this method. The number of data points required to map the interactions of multiple parameters increases by the power of the number of parameters. If *N* settings were evaluated to map the effect of a single parameter, *N* ^2^ would be required for a similar mapping of two parameters. For example, evaluating a single parameter at five settings takes about half an hour. Two parameters, with five settings each would require two and a half hours. Experiments by Bertram used eight different values for the speed and step frequency of subjects. Though he only tested 49 of the 64 possible combinations, the experiments required subjects to walk for over four hours [17]. Evaluating all the possible combinations for the 11 parameters of the BiOM [2] or the 42 parameters of the Vanderbilt leg [3] would be infeasible. Nor is such an exhaustive mapping necessarily useful. When the goal is to identify the energetically optimal parameter settings, parameter settings evaluated far from the minimum do not greatly affect the result. The surfaces that are fit to measurements are designed to match the *local* relationship between the parameters and energetic cost. For this reason, evaluating parameter settings close to the minimum would provide a more accurate estimate of the minimum location than the evaluation of settings far from the minimum. Yet, this is only possible if we have some prior knowledge about the approximate location of the minimum, or if we can estimate the location in an iterative process.

The purpose of our work is to improve the methods used to identify device parameter settings. Specifically, we propose methods that enable the faster selection of energetically optimal parameter settings. These methods rely on the the estimation of *Instantaneous Energetic Cost*[20]. *Instantaneous* Cost refers to power being used by the body at any particular instant in time. Estimates of Instantaneous Cost provide a way of characterizing the energetic requirements of various conditions without necessarily waiting for respiratory measurements to reach steady-state at each condition. This enables a real-time optimization that relies on continuously varying parameters that are updated with an actual human body in the loop.

## Methods

**Fig 1:**
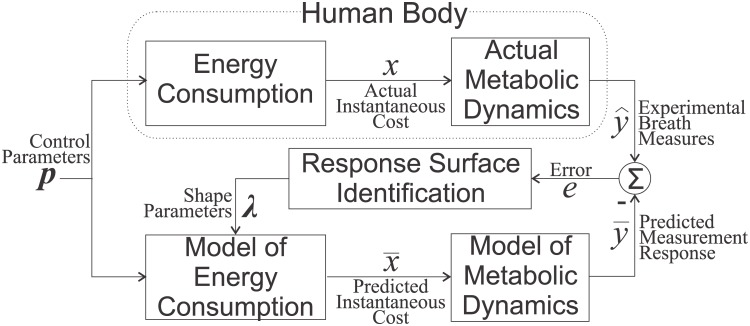
Graphical Representation of the Response Surface Identification. The relationship *x*(*p*) between a controller parameter *p* (in this work, step-frequency) and the physiological objective *x* (in this work, metabolic energetic cost) was replicated by a response surface $\bar{x}\left(p,\lambda \right)$ defined by a set of shape parameters ***λ***. The shape parameters were identified to minimize the error between predicted respiratory response $\bar{y}$ (based on a model of the measurement dynamics [20]) and actual measures $\hat{y}$. This dynamic estimation process enabled us to use non-steady-state breath-by-breath measures to approximate the relationship between the control parameter *p* and energetic cost *x*. Optimization was performed with respect to the response surface, $\bar{x}\left(p,\lambda \right)$, that approximates the energy-parameter relationship.

The optimization seeks to identify optimal parameter values ***p*** ^⋆^ that minimize a physiological value function *x* (Fig 1). This value function is estimated indirectly through measurements $\hat{y}$ of indicators ***y***. For example, we might seek to minimize the energetic effort of a person walking with an assistive device. In this example, the value function *x* is the subject’s instantaneous energetic cost, and the indicator *y* is oxygen consumption measured at the mouth ($\dot{V}O_{2}$), obtained via respiratory measurements $\hat{y}$. When we assume that a reduction of effort can be achieved by the proper tuning of controller parameters, the cost function can be expressed as *x*(***p***) in which the controller parameters constitute the free parameter vector ***p***. The current work demonstrates a faster method for approximating the optimal setting of a single parameter *p* (*Instantaneous Cost Mapping*) and a method that can search for the minimum when multiple parameters ***p*** are involved (*Instantaneous Cost Gradient Search*). These methods are compared with the traditional *Steady-State Cost Mapping* approach.

**Fig 2:**
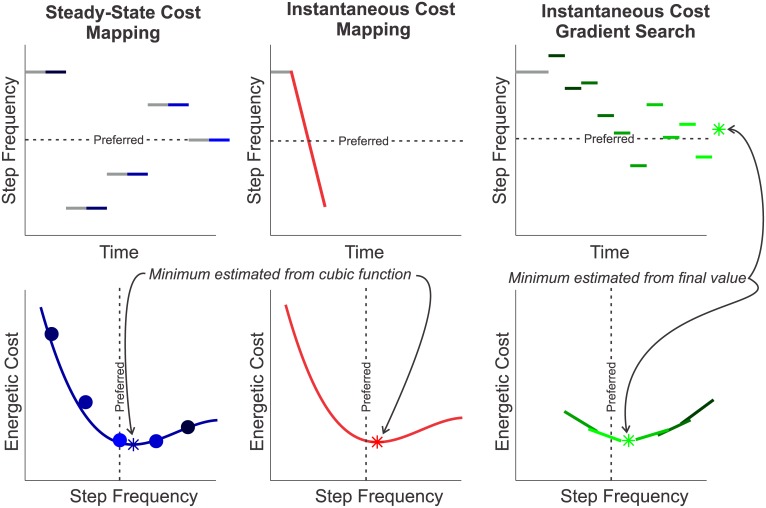
Overview of Methods. *Steady-State Cost Mapping* uses a cubic polynomial fit to averages of steady-state metabolic measurements taken in random order at discrete step frequencies between -25% and 25% of the subject’s preferred step frequency. *Instantaneous Cost Mapping* uses data collected from continuously changing the commanded step frequency over the same range. The predicted minimum of this method is obtained from a cubic polynomial response surface fit that best predicts the respiratory measurements. The *Instantaneous Cost Gradient Search* uses an estimate of the slope of the energy-parameter surface to update the guess of the optimal parameter setting. The slope estimate comes from measurements taken at discrete step frequencies a small distance away from the current guess. The predicted minimum is the final iteration value. (Gray lines indicate data that are not used in the identification.)

The estimation of Instantaneous Cost relies on a function that serves as a surrogate for the unknown relationship between energetic cost and the parameter settings. The surrogate function is adapted to match experimental measurements of energetic cost [17, 20]. This surrogate function can be interpreted as a *response surface* that characterizes the energy-parameter relationship and can be used to drive an optimization [22]. The *Instantaneous Cost Mapping* uses a cubic polynomial fit to the entire feasible range of a parameter. The measurements for the polynomial are taken while linearly varying the parameter value from one extreme to the other over a fixed time interval. The estimate of the optimal parameter setting is found by evaluating the minimum of the polynomial. The *Instantaneous Cost Gradient Search* uses gradient descent techniques adapted from Finite Difference Stochastic Approximation [23]. This method approximates a gradient using measures taken at points near the current parameter settings. The gradient (i.e., a linear response surface) is used to iteratively drive the parameter settings to a local minimum. This process takes place with a “body-in-the-loop”–it is driven by measurements of energetic cost collected in real-time. The three methods are illustrated in Fig 2.

**Fig 3:**
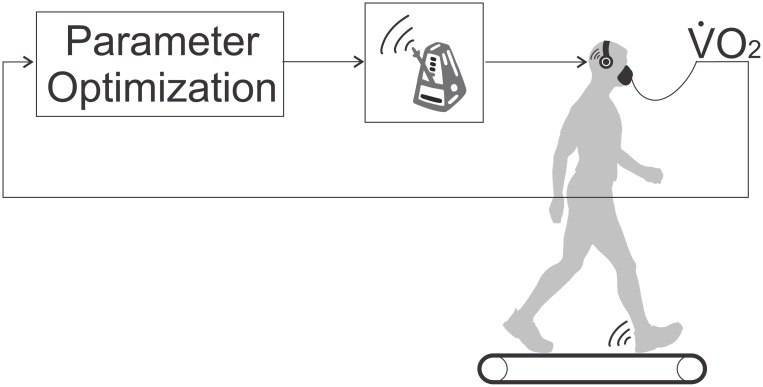
Experimental Evaluation. “Body-in-the-Loop” optimization refers to the use of real-time measures of a physiological value function to drive the online selection of parameter values. We evaluated this concept with the example of treadmill walking to the beat of a metronome. In a study with eight healthy subjects, we used step-frequency as a proxy for the controller parameters of a computer controlled assistive device. Our physiological objective was the minimization of metabolic energetic cost. Three different computer algorithms were implemented that prescribed step-frequency via the metronome and that evaluated measures of oxygen consumption.

In the study presented here, the device parameter *p* that we optimize is simply the step frequency at which a subject is commanded to walk. That is, rather than using a robotic assistive device unique to our lab, our “computer-controlled assistive device” is a metronome that subjects are instructed to follow (Fig 3). Our use of step frequency allows the methods of this work to be easily replicated and improved by other groups. Because step frequency is easy to prescribe and measure and has a clear metabolic minimum, it has been used in experiments studying the way that humans optimize their own walking [15, 17, 24]. The energetically optimal step frequency can be approximated by the step frequency that a subject selects on their own (without a metronome) [15]. This self-selected step frequency is referred to as a subject’s “preferred”. Our experiments with eight subjects used the preferred step frequency of each subject as the ground truth for the energetic minimum.

### Parameter Exploration Strategies

By exploring the parameter settings, ***p***, in an intelligent way, we can learn about the shape of the cost function, *x*(***p***), and find energetically optimal values of the parameters, ***p*** ^⋆^. The algorithms we test explore the parameters in different ways: the *Steady-State Cost Mapping* relies on evenly-spaced parameter settings evaluated in separate trials, the *Instantaneous Cost Mapping* evaluates a continuous sequence of parameter settings in a single trial, and the *Instantaneous Cost Gradient Search* tests nearby parameter settings to intelligently update the parameter choice.

### Response Surface Identification

The instantaneous energetic cost, *x*, cannot be measured directly, but only estimated through the *respiratory response*, *y*. These measurements are intermittent, very noisy and exhibit dynamics similar to a low-pass filter. Without replicating the complex biological processes that govern respiration and metabolism, we describe the dynamic relationship of *instantaneous energetic cost* *x* and the *respiratory response* *y* by a simple lumped-parameter model with experimentally identified coefficients. In particular, our previous work shows that a first-order, linear dynamic model with a single time constant, *τ*, can describe the dynamics of respiratory metabolic measurements during walking [20]. That is, the dynamic response of *y* is akin to a low-pass filter with a time constant of *τ*. In [20], this grey-box model was able to account for 82–99% of the measured variability. Mathematically, the relationship between the respiratory response of energetic cost, *y* ^*i*^ (corresponding to breath, *i*), and the instantaneous energetic cost, *x* ^*i*^(***p*** ^***i***^), can be expressed as:

$$\begin{array}{c} y^{i}=\left(1-\frac{h_{i}}{\tau }\right)y^{\left(i-1\right)}+\frac{h_{i}}{\tau }x^{i}\left(p^{i}\right). \end{array}$$ (1)

In this time-discrete dynamic formulation, *h*_*i*_ is the time since the previous breath and *τ* is the time constant of the subject’s metabolic response. If *x* and *y* were at the same value (i.e., steady-state conditions) and the value of *x* was suddenly changed, the subsequent values of *y* would begin to asymptotically approach the new value of *x*. *τ* characterizes the rate of this asymptotic approach. After a time equal to 3*τ* the error between *x* and *y* will be approximately 95% smaller than before. Previous experiments characterizing instantaneous energetic cost during walking found an average value of *τ*, in their subject pool, to be 42 seconds with a standard deviation of 12 seconds [20].

We approximate the instantaneous cost function *x*(***p***) by finding a *response surface* that best predicts the respiratory measurements while taking into account the dynamics of metabolic energy conversion. This response surface is defined by a vector of *m* constants, ***λ***. The constants alter the shape of the response surface and are identified by the algorithm to match the experimental measurements of energetic cost. We denote this approximation of the underlying relationship between the parameters, ***p***, and the instantaneous energetic cost, *x*(***p***) with an overbar: $\bar{x}\left(p,\lambda \right)$. The optimization is conducted with respect to this response surface, similar to work by Lawrence et al. [25]. While there are many functions that could be used to represent the relationship between the parameters and the required energy, the response surfaces used in this work are polynomial series with coefficients ***λ***. Polynomials are useful for approximating unknown functions because they are linear with respect to ***λ***.

Recursively evaluating Eq (1) using $\bar{x}\left(p,\lambda \right)$ and an initial breath estimate ${\bar{y}}^{1}$ returns a predicted respiratory response $\bar{y}$. For a given set of *n* noisy breath measurements, $\hat{y}$ (where ^ refers to actual measurements), the best fit response surface minimizes the sum of squared error, *e*, between the predicted respiratory response, $\bar{y}$, and the actual measurements, $\hat{y}$:

$$\underset{\lambda ,{\bar{y}}^{1}}{\text{min}}\;e=\sum_{i=1}^{n}{\left({\hat{y}}^{i}-{\bar{y}}^{i}\right)}^{2}={\left({\hat{y}}^{1}-{\bar{y}}^{1}\right)}^{2}+\sum_{i=2}^{n}{\left({\hat{y}}^{\;i}-{\bar{y}}^{i}\left(\bar{x}\left(p^{i},\lambda \right),{\bar{y}}^{i-1},h^{i},\tau \right)\right)}^{2}$$ (2)

The solution to Eq (2) includes a set of optimal shape-defining constants, ***λ*** ^⋆^, that result in a response surface, $\bar{x}\left(p,\lambda^{⋆}\right)$, with the best fit between the predicted respiratory response, $\bar{y}$, and the actual breath measurements, $\hat{y}$. This method of response surface identification is similar to Least-Squares techniques of dynamic system parameter estimation [26]. A well-fit response surface provides a way for us to characterize the effect of the parameters on the energetic cost in an analytic form.

Because our polynomial response surfaces are linear with respect to ***λ***, Eq (1) can be expressed as a system of linear equations

$$\begin{array}{c} A(\begin{array}{c} {\bar{y}}^{1}\\ \lambda_{1}\\ \vdots \\ \lambda_{m} \end{array})=(\begin{array}{c} {\bar{y}}^{1}\\ \vdots \\ {\bar{y}}^{n} \end{array}). \end{array}$$ (3)

This linear form allows us to efficiently find the optimal initial breath estimate, ${\bar{y}}^{1⋆}$, and shape parameters, ***λ*** ^⋆^ that satisfy Eq (2). This is done using the pseudoinverse of **A**, **A** ^+^ = (**A** ^*T*^ **A**)^−1^ **A** ^*T*^. The properties of the pseudo-inverse allow us to find the best-fit response surface by multiplying **A** ^+^ with the respiratory measurements $\hat{y}$. The respiratory response, $\bar{y}$, predicted by ${\bar{y}}^{1⋆}$ and ***λ*** ^⋆^ will have the least squared-error possible.

$$\begin{array}{c} \left(\begin{array}{c} {\bar{y}}^{1⋆}\\ \lambda_{1}^{⋆}\\ \vdots \\ \lambda_{m}^{⋆} \end{array})=A^{+}(\begin{array}{c} {\hat{y}}^{1}\\ \vdots \\ {\hat{y}}^{n} \end{array}\right) \end{array}$$ (4)

**A** is a (*n*) × (*m* + 1) matrix whose elements are defined recursively by the following pattern.

$$\begin{array}{c} \begin{array}{c} A_{i\;j}=\{\begin{array}{cc} 1\;\;\; & i=1\;,j=1\\ 0\;\;\; & i=1\;,j\neq 1\\ A_{(i-1)\;j}\cdot (1-\frac{h_{i}}{\tau })\;\;\; & i>1\;,j=1\\ A_{(i-1)\;j}\cdot (1-\frac{h_{i}}{\tau })+\frac{h_{i}}{\tau }\frac{\partial x(\lambda ,p^{i})}{\partial \lambda_{j-1}}\;\;\; & i>1\;,j>1 \end{array} \end{array} \end{array}$$ (5)

When using a polynomial response surface, $\bar{x}\left(\lambda ,p\right)$ for a single parameter, *p* of the form $\bar{x}\left(\lambda ,p\right)=\lambda_{1}+\lambda_{2}p+\lambda_{3}p^{2}+\ldots +\lambda_{m}p^{m-1}$, the matrix **A** is defined as follows

$$\begin{array}{cc} A= & (\begin{array}{cccc} 1 & 0 & 0 & \cdots \\ \left(1-\frac{h_{i}}{\tau }\right) & \frac{h_{i}}{\tau } & \frac{h_{i}}{\tau }p & \cdots \\ {\left(1-\frac{h_{i}}{\tau }\right)}^{2} & \frac{h_{i}}{\tau }\left(1-\frac{h_{i}}{\tau }\right)+\frac{h_{i}}{\tau } & \frac{h_{i}}{\tau }p\left(1-\frac{h_{i}}{\tau }\right)+\frac{h_{i}}{\tau }p & \cdots \\ \vdots & \vdots & \vdots & \vdots \end{array}\\ & \begin{array}{cc} \cdots & 0\\ \cdots & \frac{h_{i}}{\tau }p^{m-1}\\ \cdots & \frac{h_{i}}{\tau }p^{m-1}\left(1-\frac{h_{i}}{\tau }\right)+\frac{h_{i}}{\tau }p^{m-1}\\ \vdots & \vdots \end{array}). \end{array}$$ (6)

In the Instantaneous Cost Mapping, the response surface identification process is done only once, after the experiment is completed. In the case of the Instantaneous Cost Gradient Search, this identification process is done repeatedly during the experiment to iteratively drive the parameters towards energetically optimal values. We implemented this method in MATLAB, and have made it available on the MATLAB File Exchange [27].

### Instantaneous Cost Mapping

For the Instantaneous Cost Mapping method, we fit a cubic polynomial response surface to the parameter space by continuously changing parameter values during the experiment. The analytical minimum of the surface is returned as the optimal parameter choice.

#### Parameter Exploration in the Instantaneous Cost Mapping

The parameter space is explored by linearly changing the parameter value from one extreme to the other (e.g. *p*_*min*_ to *p*_*max*_) over the course of time *T*.

$$\begin{array}{c} p\left(t\right)=p_{min}+(\frac{p_{max}-p_{min}}{T})t \end{array}$$ (7)

Larger values of the interval, *T*, result in an increased number of samples which reduces the uncertainty of the shape parameters. Our experiments use *T* = 5 min. The algorithm begins by commanding a step frequency at either 25% above or 25% below the subjects preferred step frequency. This step frequency gradually changes over the course of five minutes to the other value (e.g., from -25% to 25%).

#### Response Surface Type for the Instantaneous Cost Mapping

We assume that the underlying relationship between the parameters and energetic cost can be approximated by a cubic polynomial function.

$$\begin{array}{c} \bar{x}(\lambda ,p)=\lambda_{1}+\lambda_{2}p+\lambda_{3}p^{2}+\lambda_{4}p^{3} \end{array}$$ (8)

The minimum of the polynomial is the unique point where the first derivative of the polynomial (with respect to *p*) is zero and the second derivative is positive.

### Instantaneous Cost Gradient Search

This method iteratively approaches the local minimum of the value function by using the gradient (i.e., slope) of the value function to update its guess of the best parameter settings.

#### Parameter Exploration in the Instantaneous Cost Gradient Search

Gradient searches work by evaluating the gradient of the energy-parameter relationship near the current parameter settings. This gradient can be thought of an arrow pointing in the direction of improvements in energetic cost. The algorithm works by following this arrow towards the minimum. For example, if one imagines an energy-parameter relationship that looks like a parabola, the gradient would always be pointing in the direction of the minimum. The general form of a gradient descent is

$$\begin{array}{c} {\bar{p}}_{k+1}={\bar{p}}_{k}-\alpha_{k}J_{k}\;\;\;\;\alpha_{k}>0 \end{array}$$ (9)

where ${\bar{p}}_{k}$ is the vector of parameter values at the current iteration, ${\bar{p}}_{k+1}$ contains the parameter values at the next iteration, **J**_*k*_ is the local gradient and *α*_*k*_ is a small gain that determines the size of the change in parameters. Larger values of *α*_*k*_ can be used to improve the progress of the algorithm far from a minimum and smaller values can be used to improve the convergence of the algorithm near a minimum. This method assumes that the cost function is differentiable with respect to the parameters. **J**_*k*_ at the current parameter settings ${\bar{p}}_{k}$ is a vector containing the local slopes of the value function with respect to each parameter (*p*_1_, *p*_2_, *p*_3_ etc.).

$$J_{k}=\nabla {\left(x\right)}_{p}={\left(\begin{array}{c} \frac{\partial x}{\partial p_{1}}\\ \frac{\partial x}{\partial p_{2}}\\ \frac{\partial x}{\partial p_{3}}\\ \vdots \end{array}\right)|}_{{\bar{p}}_{k}}$$ (10)

For a value function evaluated through measurements, one cannot evaluate the gradient directly. An estimate of the gradient can be found by fitting a linear response surface to parameter values near the current iteration. Because of the uncertainty propagation from measurement noise, this estimate will be a stochastic approximation of the true gradient. Finite-Difference Stochastic Approximation (Kiefer-Wolfowitz) [23] optimization techniques provide a template for identifying and utilizing these uncertain gradients in the search for a minimum. These methods estimate gradients by perturbing parameters with a “finite difference” and observing the response. The estimates of the gradients are used with a “scheduled” gain, *α*_*k*_ that decreases with as the iteration number, k, increases according to the following equation.

$$\begin{array}{c} \alpha_{k}=\frac{A_{0}\alpha_{0}}{A_{0}+k^{\gamma }}\;\;\;\;\;\;\;0<\gamma \leq 1 \end{array}$$ (11)

In this method, measurements are taken at a perturbation distance, *c*_*k*_, from the current iteration point, ${\bar{p}}_{k}$. In our experiments, *c*_*k*_ was kept at the constant at 5% of the subjects preferred step frequency. The parameters of the gain schedule were *A*_0_ = 3, α_0_ = .0004^*hz*^2^^/_ml/min_, γ = 1. To estimate the gradient in this one-dimensional case with *n* measurements of energetic cost, the parameter value was first perturbed upwards for $\frac{n}{2}$ breaths and then downwards for another $\frac{n}{2}$ breaths. *i* is the number of breaths since the gradient fitting began. In our experiments, we evaluated the gradient with 30 breaths at each perturbation point (i.e., *n* = 60).

$$\begin{array}{c} \begin{array}{c} p_{i}=\{\begin{array}{cc} {\bar{p}}_{k}+c_{k}, & \text{if}\;\;i\leq \frac{n}{2}\\ {\bar{p}}_{k}-c_{k}, & \text{if}\;\;\frac{n}{2}<i\leq n \end{array} \end{array} \end{array}$$ (12)

#### Response Surface Type for the Instantaneous Cost Gradient Search

A linear response surface is fit to the set of *n* measurements of energetic cost taken near the current iteration point, ${\bar{p}}_{k}$. This surface allows us to extract an estimate of the gradient, $J_{k}=\lambda_{2}^{⋆}$.

$$\begin{array}{c} \bar{x}(\lambda ,p)=\lambda_{1}+\lambda_{2}p \end{array}$$ (13)

### Experimental Methods

We implemented the three algorithms and evaluated their performance with eight healthy subjects walking to the beat of a metronome. Table 1 provides subject metrics. Subjects provided, written informed consent according to procedures approved by the University of Michigan Institutional Review Board. The methods of the study were approved prior to the commencement of the study by the IRB listed below. Study eResearch ID: HUM00020554 Health Sciences and Behavioral Sciences Institutional Review Board (IRB-HSBS) 2800 Plymouth Rd., Building 520, Room 1170, Ann Arbor, MI 48109-2800 (734) 936-0933 irbhsbs@umich.edu.

**Table 1:** Subject-Specific Data. The subject’s preferred cadence (step frequency) was evaluated while on a treadmill without a metronome. “Time constant” refers to the time constant of the metabolic response used in Eq (1). This was characterized with a step-change in energetic requirements during the first six minutes of the Instantaneous Cost Mapping trial.

#	Age	Sex	Weight (kg)	Height (cm)	Preferred Cadence (Hz)	Time Constant (s)
S1	25	M	80	184	1.79	31
S2	20	M	77.7	178	1.75	33
S3	24	M	85	189	1.67	25
S4	19	M	88	185	1.77	15
S5	25	M	72	175	1.82	27
S6	20	F	57.7	167	1.93	34
S7	21	M	54	167	1.85	12
S8	17	F	49.1	164	1.84	47
**mean**	21		70.4	176	1.80	28
SD	3		14.9	9	0.08	11

We evaluated each subject in two sessions with no more than 10 days between sessions. We asked subjects to fast for at least two hours before each session to ensure accurate metabolic measurements. All walking was at 1.25 m/s on a Woodway PRO 27 treadmill (WOODWAY USA, Inc., Waukesha, WI, USA). We conducted the Steady-State Cost Mapping in the first session. The Instantaneous Cost Mapping and Instantaneous Cost Gradient Search were conducted in this order in the second session. The time constant of each subject’s metabolic response was identified during the Instantaneous Cost Mapping method.

We measured oxygen consumption with a portable respirometer (*K*4*B* ^2^, COSMED, Rome, Italy). Oxygen consumption, $\dot{V}O_{2}$, was taken as a proxy for metabolic energetic cost. Energetic cost values calculated with oxygen consumption alone will not differ by more than 3% from energetic cost values calculated with both oxygen consumption and *CO*_2_ production [19]. The step-frequencies were commanded through an Arduino-controlled metronome and measured with a force-sensitive-resistor (FSR) under the first metatarsal of the right foot. The FSR sensor was attached to a wireless transmission system (Trigno Wireless EMG, DELSYS, Natick, MA, USA). The measured step-frequency was filtered with a four-foot-fall (of the right foot) moving average filter. The filter was designed to roughly associate each breath with the contemporaneous step frequency measurements. Given a typical breath-rate and cadence of 20 ^breaths/minute^ and 1.8 Hz, respectively, we would expect three foot-falls of the right foot between breaths. From these filtered values, step frequencies concurrent to the recorded breaths were extracted through linear interpolation and used for processing. All algorithms were implemented in MATLAB (The MathWorks, Inc., Natick, MA, United States). We developed custom scripts to extract real-time $\dot{V}O_{2}$ data from the COSMED software and real-time step frequency from the EMG software into MATLAB.

In the Steady-State Cost Mapping, subjects first stood at rest for six minutes so we could evaluate their resting metabolic rate. We then asked the subjects to “walk normally” for six minutes. We averaged the last three minutes of measured step frequencies to establish the subject’s preferred step frequency. We then evaluated subjects at step frequencies commanded by the metronome between 25% above and 25% below their preferred step frequency at 5% intervals in a random sequence (including an evaluation at 0%, i.e., with the subject’s preferred step frequency commanded by the metronome). In each condition, we allowed three minutes to reach steady-state before averaging the measurements of oxygen consumption over an additional three minutes at the condition. Waking bouts were preceded by rests and did not last more than 18 minutes. We again evaluated subjects standing at rest for six minutes. A third-order polynomial was fit to the means of the steady-state recorded step frequencies and $\dot{V}O_{2}$. The minimum of this polynomial was taken as an estimate of the true energetic minimum.

For the Instantaneous Cost Mapping, subjects stood at rest for three minutes, then walked for three minutes at a starting cadence randomly selected to be either 25% above or below their preferred cadence. This data was used to identify the time-constant of the subject’s metabolic dynamics. Since our previous work found no significant difference between time constants for increases and decreases in activity level [20], we did not evaluate an additional, decreasing step. In order to identify the time constant, we needed to approximate the true instantaneous cost underlying the measures. We approximated the instantaneous cost as a step input that moves from a standing rest value up to a starting cadence value. For the standing rest value, we averaged the respiratory measurements from the last minute at that condition. For the starting cadence, we also used an average of the last minute of data. With this definition of instantaneous cost, *x*, MATLAB’s optimization toolbox was used to search for the value of *τ* and ${\bar{y}}^{1}$ that minimized the sum of the squared error between the predicted dynamic response, $\bar{y}$, and the measurements,$\hat{y}$, taken during the entire first six minutes of the test. This process was adapted from our prior work [20]. These six minutes were followed by a metronome beat which moved from the starting cadence to the other extreme over the course of five minutes (i.e., moving from -25% to +25% or vice versa). Using the time constant we identified from the first part of the test, we used the data from the last five minutes of the test to identify the best-fit cubic polynomial response surface. The minimum of this polynomial was taken as an estimate of the true minimum.

In the Instantaneous Cost Gradient Search, the starting condition was randomly chosen to be either 20% above or below the subjects preferred cadence. Subjects began by walking for three minutes at the starting condition to reach steady-state before the optimization commenced. The data acquired during this interval was not used in the optimization. The gradient was evaluated using 60 breaths (approximately 3 minutes). The perturbation width was 5%. For example, if the starting condition was 20% above the subject’s preferred step frequency, after warming up at the starting condition, the algorithm first commanded a metronome beat 25% above the subject’s preferred value for 30 breaths and then commanded a beat at 15% for 30 breaths. The algorithm then iterated along the gradient according to Eq (9) and repeated the gradient evaluation process. At any point or after five iterations, the optimization was stopped and the subject was allowed to rest as desired. When the test resumed, the algorithm again waited three minutes with the subject walking at the parameters defined by the last iteration before evaluating the gradient again. The algorithm was terminated after 15 iterations. The choice of the number of breaths, *n*, and the gain schedule were evaluated in simulation to verify their performance under the noise conditions. This algorithm assumed the subject’s time constant to be forty seconds [20]. The estimated minimum was taken to be the termination point of the algorithm.

## Results

**Fig 4:**
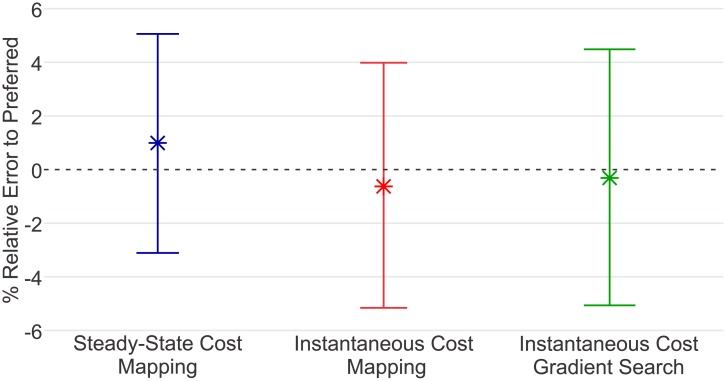
Accuracy of the Three Methods. Shown are means and standard deviations of the predicted energetic minima. We use the subjects’ preferred step frequency as ‘ground truth’ for comparison [15]. All three methods, on average, identify energetic minima near the subjects preferred cadence. The variance in the outcomes of the proposed Instantaneous Cost methods is only slightly higher than that of the established Steady-State Cost Mapping. There is no significant difference between the absolute error of the three methods.

**Fig 5:**
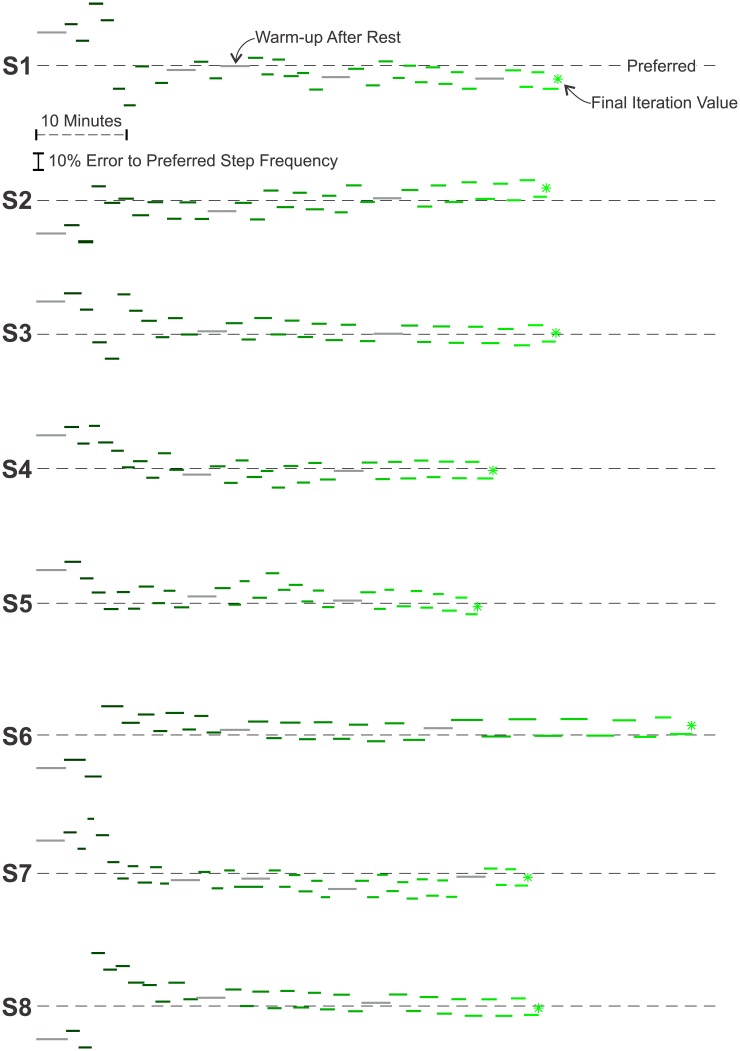
Convergence of the Instantaneous Cost Gradient Search Method. The lines indicate the prescribed step frequencies over the course of the experiment. The experiment begins with an incorrect guess of the optimal step frequency that is randomly selected to be either 20% above or below the subject’s preferred step frequency. After a warm-up period, the step frequency is perturbed above and below the guess. The measurements of oxygen consumption during this perturbation process are used to estimate the slope of the underlying relationship between energetic cost and step frequency. This slope, or gradient, is used to update the guess of the optimal step frequency. Though the performance of the algorithm varied between subjects, the algorithm was always able to approach the energetic minimum. The average total walking-time was 56.5 minutes and the mean relative error of the final iteration value to the subject’s preferred step frequency was −0.29% ± 4.77% (mean ± SD). The lightening of shades indicates the progress of time.

**Fig 6:**
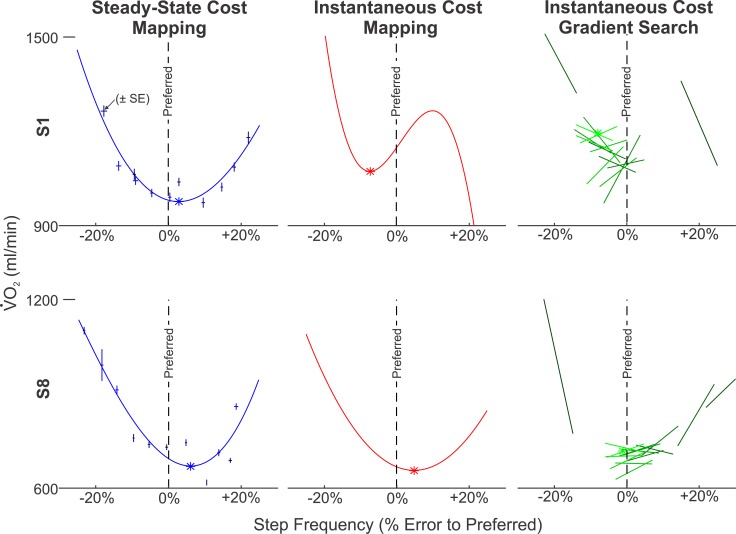
Examples of Response Surface Estimates of the Three Methods. Data are shown for Subject 1 and Subject 8 (poor and good performance of Instantaneous Cost methods respectively). This figure illustrates the third-order polynomial fit to the means (± SE) in the Steady-State Cost Mapping method, the third order polynomial response surface that best predicts the measures from the Instantaneous Cost Mapping, and the series of linear response surfaces used to update the parameter guess in the Instantaneous Cost Gradient Search (the lightening of shades indicates the sequence of the fits). The linear response surfaces of the Instantaneous Cost Gradient Search for Subject 1 show a much higher degree of variability than Subject 8. This led the algorithm to converge at a value somewhat below the subject’s preferred step frequency (worse than any other subject). Interestingly, the Instantaneous Cost Mapping for Subject 1 estimated a similarly low minimum. For subject 8, the linear response surfaces are more regular, leading the gradient search algorithm to outperform both of the mapping methods.

The minima predicted by the three algorithms are listed in Table 2 and illustrated in Fig 4. The Steady-State Cost Mapping required 66 minutes of walking. The minima of the resulting curves had an average relative error of 0.975% ± 4.08% (mean ± SD) with respect to the subjects’ preferred step frequencies. The Instantaneous Cost Mapping required only 8 minutes of walking. The time constants of our subjects had an average value of 28 sec ± 11 sec (mean ± SD). The minima of the of the resulting cubic polynomials had an average relative error of −0.59% ± 4.57% (mean ± SD) with respect the subjects’ preferred step frequencies. After fifteen iterations, the final conditions of the Instantaneous Cost Gradient Search had an average relative error of −0.29% ± 4.77% (mean ± SD) with respect to the subjects’ preferred step frequencies. The average walking-time for this method was 56.5 minutes. Fig 5 illustrates the progress of this algorithm for each subject. Fig 6 compares the results of the three methods for two representative subjects. The absolute error for each method was 3.41% ± 2.11% (mean ± SD) for the Steady-State Cost Mapping, 3.92% ± 2.89% (mean ± SD) for the Instantaneous Cost Mapping and 3.56% ± 1.92% (mean ± SD) for the Instantaneous Cost Gradient Descent.

**Table 2:** Optimization Results. Listed are estimates of the energetic minima resulting from the different algorithms. Values are in percent-error with respect to the subject’s preferred step frequency. SSCM refers to a Steady-State Cost Mapping with a best-fit third-order polynomial. ICM refers to the minima of the Instantaneous Cost Mapping. ICGS refers to terminal values of the Instantaneous Cost Gradient Search.

#	SSCM	ICM	ICGS
S1	2.82%	-7.35%	-7.97%
S2	1.41%	-0.54%	7.1%
S3	1.19%	4.51%	0.56%
S4	-5.54%	-3.70%	-1.08%
S5	6.00%	-2.89%	-2.23%
S6	-2.18%	3.96%	5.36%
S7	-2.02%	-3.57%	-2.93%
S8	6.12%	4.88%	-1.21%
**mean**	0.975%	-0.59%	-0.29%
SD	4.08%	4.57%	4.77%

## Discussion

These methods represent a valuable new tool for the selection of parameters in devices that interact with the human body. Our experiments demonstrate that the optimization algorithms we propose are robust to variations between subjects. The means of the error of the minima predicted by the three methods are each within a standard error (SE) from 0%. This means that none of the methods exhibited a significant bias towards selecting minima above or below the subjects’ preferred step frequencies. The accuracy of a particular method can be characterized by the variance of the estimated minima. A smaller variance would suggest that the method is more likely to select minima close to the true energetic minimum. In this sample, the Steady-State Cost Mapping has a slightly smaller variance than the other two methods. However, a Levene test for homogeneity of the variances does not show the variances to be significantly different (*p* = 0.88) [28]. Moreover, an F-test on the absolute error of the methods does not reveal any significant difference (*p* > 0.49). This suggests that methods proposed in this work can identify energetically optimal parameters settings with similar accuracy to traditional methods.

### Faster Minimum Identification

For the experimenter, Steady-State Cost Mapping is by far the simplest method to implement. In this method, all of the data is post-processed and discrete parameter settings can be evaluated in completely separate trials (which permits parameters to be changed offline). This method, however, is time consuming and fatiguing for subjects. The Instantaneous Cost Mapping, on the other hand, can be much faster than Steady-State Cost Mapping. The implementation of this method, however, requires the parameters to be varied during the test. Like the Steady-State Cost Mapping, this method assumes a function shape for the underlying energy-parameter relationship with the assumption that the minimum of this function will be near the true energetic minimum. For clinical populations that fatigue quickly, the Instantaneous Cost Mapping provides the means to estimate energetic minima without excessive walking-time.

### Global vs Local Methods

When there are many interacting parameters to be tuned, gradient based optimization methods, such as the Instantaneous Cost Gradient Search are more efficient than mapping methods. Rather than map the entire parameter space, gradient searches iteratively improve parameter values relative to the current setting. If one imagines a parameter-cost relationship as a terrain with peaks and valleys, a gradient search algorithm can be thought to “flow” downwards towards the lowest point. Instead of blindly sampling the entire parameter space, the algorithm only needs to evaluate parameter settings along its path, increasing algorithmic efficiency. As a downside, gradient searches are “local” methods, meaning they can only find parameter combinations that reduce metabolic cost local to the starting conditions. If two valleys are separated by a ridge, the algorithm is unable to move between them. When the starting point is in the basin of the lowest valley (i.e., somewhat close to the global minimum), the algorithm will be able to reach the best-possible parameter setting. It is difficult to state whether the local nature of gradient searches poses a problem. It has been shown, for example, that the energetic cost relationship to power and timing parameters in an assistive ankle orthosis is well-characterized by a single valley [29]. For new devices, however, the shape of the cost landscape is always unknown a priori. The importance of the starting point choice depends on whether the cost landscape contains a single or multiple valleys.

### Algorithm Parameter Selection

The performance of the algorithms presented in this work can be affected by a poor choice of the parameters governing their operation. When the noise in the measurements of energetic cost is large compared to the slopes of the response surface, algorithm performance will be negatively impacted. In general, adjusting the parameters to lengthen the duration of the testing time should improve performance.

For instance, the Steady-Sate Cost Mapping works best when the data is averaged over a sufficient time. The more samples that are collected at each steady-state condition, the greater the confidence in the resulting mean. If we assume the underlying measurements come from a normal distribution, we would expect the standard error of the calculated means to have an inversely proportional relationship to the square-root of the number of samples, *n*.

The Instantaneous Cost Mapping method can be improved by increasing the duration of the parameter sweep. A slow parameter sweep reduces the attenuation in the metabolic dynamics. A quick parameter sweep may result in a rapidly changing instantaneous energetic cost. These rapid changes in cost will be will be attenuated by the low-pass-filter-like dynamic response of the breath-to-breath measures. Additionally, an increased number of samples helps to average-out the noise. If the parameter sweep is slow, the shape of the estimated energy-parameter relationship will be driven by a more accurate mean of the noisy signal.

The order of the Instantaneous Cost Mapping polynomial can also be adjusted to improve algorithm performance. The order of the polynomial can be decreased to “smooth” the effect of the noise. Doing so, however, may also hide important features of the underlying energy-parameter relationship that could be captured by higher-order approximations. For example, quadratic approximations are symmetric and only have one maximum or minimum. Higher-order approximations could be used to capture asymmetry or additional minima or maxima. These higher-order fits, however, are more-likely to be affected by noise. The negative impact of the noise would be most pronounced when using a relatively quick parameter sweep.

The Instantaneous Cost Gradient Search has many parameters than can be tuned to improve performance or adapt the algorithm. Increasing the perturbation width, *c*, and number of breaths measured at each evaluation perturbation point can improve the estimate of the gradient. Large perturbation widths, however, hurt the “local” nature of the gradient estimate. The gain schedule and number of iterations have a more complex effect on the algorithm performance. The gain schedule can be shaped to implicitly assume that the algorithm will start far from the minimum and will approach the minimum with some approximate rate. At early iterations, the evaluation point is assumed to be far from the minimum and the algorithm has the authority to take large steps. At later iterations, the algorithm may be closer to the minimum where the gradient is difficult to estimate and where large steps may cause the algorithm to overshoot. The decrease in gain with increasing iterations helps limit the step size as the algorithm approaches the minimum.

### Scaling to Multiple Parameters

Neither the Steady-State Cost Mapping nor the Instantaneous Cost Mapping readily scales to the optimization of multiple parameters simultaneously. A Steady-State Cost Mapping method that uses *N* evaluation points with a single parameter would require *N* ^*d*^ evaluation points with multiple parameters (where *d* is the number of parameters). For example, our Steady-State Cost Mapping was performed over 11 different step frequencies. This required a little over an hour of walking. If we were to attempt to identify the optimal combination of these 11 step frequencies with 11 different values of a related parameter, such as walking velocity, subjects would need to walk for over 12 hours. If it would take 12 hours of walking to map two parameters, one can imagine the difficulty of mapping the combinations of the tens of parameters used in powered prostheses [2, 3]. Using the techniques of the Instantaneous Cost Mapping the parameter values could be varied continuously, but even this method relies on a exhaustive sampling of the parameter space. The response surface will be most accurate near parameter combinations that have been tested. Without a priori knowledge of the minimum location it would be necessary to spread the experimental evaluations over all the possible combinations of parameters. Of our three methods, the Instantaneous Cost Gradient Search is the best suited method for use with high numbers of parameters. The number of required perturbations to fit the local response surface scales only linearly with the number of parameters. In our experiments, each of the 15 evaluations the gradient for the single parameter required about three minutes. If we were to repeat our experiments with multiple parameters, evaluating the gradient with our current methods would require an additional three minutes of walking for each additional parameter.

### Time Constant Sensitivity

The Instantaneous Cost methods are somewhat sensitive to errors in the estimate of the subject’s metabolic time constant. For example, when parameters are increased from one extreme to the other in the Instantaneous Cost Mapping, errors in the time-constant will result in an error in the predicted “phase lag” between the respiratory measurements and the underlying change in cost. To compensate for this effect, one could vary the parameter from one extreme to the other and then back again in a single trial. In contrast, the sensitivity of the Instantaneous Cost Gradient Search to errors in the estimation of the time constant is only moderate because errors in the time constant typically only result in errors of the magnitude of the gradient (not the sign of the gradient). When observing respiratory measurements immediately after a step change in the underlying cost, the gradient is essentially estimated by observing the *rate* of change in the breath measurements. This rate is determined by both the difference between the currently measured cost and the steady-state cost (*λ*_1_−*y* ^*i*−1^) and the ratio of the gradient to the time constant ($\frac{\lambda_{2}}{\tau }$). This is apparent when we rewrite Eq (1) in terms of the linear response surface defined in Eq (13)

$$\begin{array}{c} (y^{i}-y^{i-1})=\frac{h_{i}}{\tau }(\lambda_{1}+\lambda_{2}p-y^{i-1})=\frac{h_{i}\lambda_{2}}{\tau }p+\frac{h_{i}}{\tau }(\lambda_{1}-y^{i-1}). \end{array}$$ (14)

If there is an error in the magnitude of the time constant, it will result in an error in the magnitude of the estimated gradient, *λ*_2_. The effect of an error in the magnitude of the gradient is something akin to an error in the magnitude of the gain, *α*_*k*_. This gain does not need to have a precise magnitude for convergence to occur. For this reason, the algorithm did not rely on an individually characterized time constant for each subject. As it turned out, the average of our individually-identified time constants (28 seconds) was lower than the average-time constant of previous studies [20] on which we based the time constant used the Instantaneous Cost Gradient Search (40 seconds). This did not impede convergence.

### Subject Adaptation

Compared to the other methods, the Instantaneous Cost Gradient Search is the most robust to subject adaptation. The method relies only on recent measurements. This allows the algorithm to be stopped and restarted at any point. Thus allowing its implementation to be spread out over multiple sessions or days as needed. This property could also be beneficial if subjects are unfamiliar with a particular set of device parameters. This unfamiliarity may cause a subject to use their muscles to work against the assistance provided by the device. Such a suboptimal walking strategy will result in higher measures of energetic cost. In the Steady-State Cost Mapping or Instantaneous Cost Mapping, these higher measures would result in a response surface that is higher in that region. Given enough time, however, subjects might adapt to unfamiliar conditions and walk in an energetically improved way [24]. With the Instantaneous Cost Gradient Search, one can imagine a situation where the algorithm approaches a set of parameter settings that are unfamiliar to the user. The measured energetic cost will be skewed upward by the suboptimal walking strategy employed by the subject. The gradient, however, will not necessarily be affected by this unfamiliarity. Improved parameter setting choices will still result in a *relative* improvement in energetic cost (even when the *absolute* cost is skewed by the unfamiliarity). This relative improvement in energetic cost can still be used to direct the search.

## Conclusions

In this work, we have demonstrated novel methods to automatically identify parameters of assistive devices via the optimization of a desired physiological objective. To prove the validity of our ideas, we tested the performance of our our algorithms by manipulating the step frequency of eight subjects. These experiments confirmed that our algorithms were able to identify energetic minima (which correspond to subjects’ preferred step frequency) with similar accuracy to the traditional method of Steady-State Cost Mapping. Our methods enable considerable time savings and readily extend to multi-dimensional parameter spaces. To the best of our knowledge, the presented Instantaneous Cost Gradient Search method is the first time that an algorithmic, online optimization of energetic cost was performed with the human body in the loop. Table 3 summarizes the qualitative differences between our algorithms and traditional methods.

**Table 3:** Qualitative Comparison of the Three Methods. SSCM refers to the traditional method of Steady-State Cost Mapping. Our methods rely on the estimation of Instantaneous Cost. ICM refers to the Instantaneous Cost Mapping and ICGS to the Instantaneous Cost Gradient Search.

	SSCM	ICM	ICGS
Approximate walking-time (one parameter)	1 hr	8 min	1 hr
Requires real-time energetic data	No	No	Yes
Requires real-time parameter variation	No	Yes	Yes
Type of minimum identified	Global	Global	Local
Assumes shape of cost function	Yes	Yes	No
Time constant sensitivity	Very low	High	Moderate
Usefulness with many coupled parameters	Very Low	Limited	High
Allows for dynamic subject adaptation	No	No	Yes

The presented step-frequency study provides an interesting proxy for assistive devices. Commanding step-frequency is inherently safe and its simplicity enables an easy implementation. Furthermore, the existing literature on the relation of energetics and step-frequency allows a rigorous assessment of our results. The final goal of our work, however, is the application of the same methods in the tuning of powered prostheses, orthoses, and exoskeletons. When using energetic cost as the physiological objective, the necessary algorithmic adaptions will be minor. Although assistive devices may introduce questions about adaptation and local minima, they have a distinct advantage over optimizing step frequency. Humans are poor “computer-controlled devices”. The algorithms in this work cannot guarantee that the “commanded” step frequency was followed precisely by the subject. In fact, Fig 6 reveals large differences between the commanded and measured frequency. As a consequence, the algorithms were forced to rely on noisy measurements of step frequency. In contrast, with an assistive device the parameters would be firmly under control. In particular, we observed a bias towards walking at the preferred step frequency. It is conceivable that this improves the performance of the mapping methods because the data set becomes denser closer to the optimum (and thus in the range where it is important). We do not believe that this effect improves the Instantaneous Cost Gradient Descent, as this method is trying to evaluate a local gradient rather than estimating the global minimum. The primary challenge in the implementation would be to extend the optimization to multiple dimensions and to guarantee patient safety. As these algorithms develop, they will find increasing use for devices where traditional tuning methods are infeasible. In the long run, we do not see our approach being limited to energetic cost as the objective. Using similar methods, one can, for example, optimize gait symmetry by evaluating kinematic sensor information or enforce a desired training effort by evaluating inverse dynamics or EMG data. While such objectives will require different methods for sensor data processing, the underlying algorithmic principles will be very similar.

## Supporting Information

S1 File Data Summary. This excel file contains the data presented in the figures.
(XLSX)
